# Surveillance Technologies and Constitutional Law

**DOI:** 10.1146/annurev-criminol-030421-035102

**Published:** 2022-09-02

**Authors:** Christopher Slobogin, Sarah Brayne

**Affiliations:** 1 Vanderbilt Law School, Vanderbilt University, Nashville, Tennessee, USA; 2 Department of Sociology, University of Texas, Austin, Texas, USA

**Keywords:** surveillance, technology, crime, constitutional law, Fourth Amendment, search

## Abstract

This review focuses on government use of technology to observe, collect, or record potential criminal activity in real-time, as contrasted with “transaction surveillance” that involves government efforts to access already-existing reviews (Brayne 2018, Ridgeway 2018). Even so limited, surveillance technologies come in many guises, including closed-circuit television, automated license plate and facial readers, aerial cameras, and GPS tracking. Also classifiable as surveillance technology are devices such as thermal and electromagnetic imagers that can “see” through walls and clothing. Finally, surveillance includes wiretapping and other forms of communication interception. The following discussion briefly examines the limited evidence we have about the prevalence and effectiveness of these technologies and then describes the law governing surveillance, focusing principally on constitutional doctrine, and how it might—and might not—limit use of these technologies in the future.

## INTRODUCTION

The typical urban police department today makes daily use of an array of real-time surveillance tools, ranging from closed-circuit television (CCTV), aerial cameras, and cellphone tracking to automatic license plate readers (ALPRs), facial recognition technology (FRT), and gunshot detection systems. Despite their popularity, the effectiveness of these surveillance technologies in achieving the government’s aims is largely unstudied, as is their empirical validity and reliability. Furthermore, their use has not been subject to the type of constitutional and statutory regulation that has long governed use of other types of real-time surveillance, such as wiretapping and bugging.

This article first reviews the small amount of existing research on the efficacy of these surveillance technologies. It then canvasses the extent to which the constitutional law—and, to a lesser extent, statutory and evidentiary law—has or might put strictures on their deployment. The focus of this review is on real-time surveillance, as opposed to the use of computers, algorithms, and other technologies to access and analyze already-collected data.

## PREVALENCE AND EFFECTIVENESS OF SURVEILLANCE TECHNOLOGIES

Police use surveillance technologies for a wide variety of purposes, ranging from deterring or intercepting crime to suppressing protest and collecting data for future investigations. Yet we know surprisingly little about the surveillance capacities of police departments nationwide. There is little in the way of a basic descriptive inventory of the surveillance technologies local and federal law enforcement agencies use, and there is even less in the way of peer-reviewed, independent evaluations of the effectiveness or impact of these technologies in terms of crime reduction, accuracy, error rates, equity, or cases cleared by arrest.

Methodologically, a key reason for this paucity of information is that these technologies are usually not rolled out in any sort of systematic way, making it difficult to make causal inferences about their impact. More fundamental, however, is that unless one works with law enforcement or the private companies that develop these programs, access to relevant data is limited, which precludes any sort of independent oversight, scientific evaluation, or community input. Owing to the lack of external evaluations, legal scholars and judges rely heavily on company-produced information on their prevalence, use, reliability, and validity, which may overstate the utility of technologies or understate problems or unintended consequences.

There are various reasons for the lack of transparency around surveillance technologies. Some of the secrecy is by design—law enforcement does not want to disclose cutting-edge investigative techniques (Hill 2021). The police may even be prevented from doing so by trade secret law or nondisclosure agreements negotiated as part of the procurement package if, as is usually the case, the technology is developed by private companies (Bloch-Wehba 2021, Wexler 2018). Even in the absence of formal barriers, law enforcement administrators themselves may not be aware of how their subordinates are using technology (see description of FRT below). Moreover, law enforcement often unevenly implements new surveillance technologies, potentially resulting in unequally distributed collateral consequences and privacy concerns (Brayne 2021) that police forces may want to hide from the general public.

Despite the lack of systematic data, researchers and journalists have begun to open the black box of police departments’ surveillance technologies through qualitative case studies (e.g., see Brayne 2021, Crump 2016, Snyder 2020), surveys (e.g., see Adams & Mastracci 2019, Gettinger 2020, LEMAS 2016, Lum et al. 2019, Oliver & Kugler 2021), and public records requests (Atlas Surveill. 2021). Below is a nonexhaustive overview of the most pervasive surveillance technologies used by law enforcement and the scant evidence on their prevalence and efficacy.

### Automatic License Plate Readers

ALPRs capture machine-readable images of license plates. These high-speed cameras may be mobile (e.g., mounted on police cars) or stationary (e.g., mounted on road signs, traffic lights, or bridges) and can capture thousands of plates a minute. Cameras typically capture two photos of every vehicle that passes through their line of vision—one of the car and one of the license plate— and record the location, date, and time the plate was captured. These data then can be linked to and compared with information in other databases, including traffic and toll cameras or “heat lists” of outstanding warrants or stolen cars (Brayne 2021). Law enforcement–collected ALPR data can also be supplemented with privately collected ALPRs, such as those used by repossession agents. The most common use of ALPRs is simply to store data for potential use during a future investigation. For example, if a body is found, law enforcement can look for nearby ALPR readings around the time the body is thought to have been left. The data produced by ALPRs can also give the police a map of the distribution of vehicles throughout the city and, in some cases, may enable law enforcement to see a given driver’s typical travel patterns or infer where they are staying based on where a vehicle is parked overnight (Brayne 2021).

ALPRs are becoming increasingly common. According to a recent national survey of law enforcement agencies (Oliver & Kugler 2021), almost two-thirds of larger police agencies—those with 100 or more officers—use ALPRs. Jurisdictions that are more urban and have a lower percentage of non-Hispanic White residents are more likely to use ALPRs (Oliver & Kugler 2021, appendix B).

Despite their spread, the evidence base for the effectiveness of ALPRs is extremely limited. In their review of the research, Prince et al. (2021, p. 695) found that ALPRs have only “modest positive impacts on case clearances for auto-theft and robbery” and that “large numbers of license plate readers are required to create those modest impacts.” Similarly, Lum & Koper (2017, pp. 121–22) concluded that “there is very limited evidence on whether LPR use actually reduces auto theft or other crimes and what types of LPR uses best achieve those ends” and noted that the available studies as of 2017 were of “short duration” or “low dosage.” Their own subsequent study (Lum & Koper 2019) found that case clearances for auto theft and robbery increased after the installation of an LPR network but not to a statistically significant degree. Furthermore, ALPR accuracy is suspect. Even a police-conducted study of ALPRs found an error rate of more than 35% (Potts 2018).

### Vehicle Tracking Devices

Whereas ALPRs are dragnet surveillance tools, meaning they collect information on everyone rather than merely those under suspicion, there are also vehicle tracking devices that law enforcement can embed in specific target vehicles. These devices are used to provide remote tracking capability when line-of-sight tracking is not feasible.

GPS devices embedded in vehicles have become increasingly common in both small and large police departments, with almost one in four law enforcement agencies reporting some reliance on them. They are relatively cheap and easy to use. Adoption follows a pattern similar to that found with ALPRs; agencies in more urban areas and areas with lower percentages of non-Hispanic Whites are more likely to use vehicle tracking devices than rural areas or those with predominantly non-Hispanic White residents (Oliver & Kugler 2021).

A 2017 report from the National Institute of Justice suggests the efficacy of using vehicle trackers as a pursuit technology varies widely by department, in part because it depends on how well they are implemented. In May 2015, the Office of Community Oriented Policing Services (Martinez 2015), a component of the US Department of Justice, analyzed GPS tracking in Redlands, CA, and found that GPS tracking “resulted in 140 arrests for crimes such as armed robbery, commercial burglary, vehicle burglary, laptop theft, bike theft, metal theft, and theft from a cemetery.” However, the report did not provide any counterfactual comparison or data about how many arrests would have been made without the technology. It also stated, “the reality is that most deployments do not result in apprehensions” (Martinez 2015).

### Aerial Surveillance

Aerial surveillance, although more expensive and less common than vehicular surveillance, is a growing modality of law enforcement surveillance. Aerial surveillance can be conducted via plane, helicopter, or unmanned aerial vehicle (UAV, or drone).As with ALPRs and tracking devices, aerial surveillance can be conducted in real-time or record images for later access.

The Center for the Study of the Drone at Bard College found that as of 2020, 559municipal police departments in the United States had acquired drones (Gettinger 2020). The Center hypothesizes that this figure may be an underestimate, as departments often share or contract out the use of drone services. In their nationwide survey, Oliver & Kugler (2021) found that 26.8% of the surveyed departments overall and 45.0% of jurisdictions with more than 10,000 residents use drones.

There have been reports of jurisdictions in Illinois, Ohio, and California claiming UAVs helped them more quickly document accidents and reopen roadways, find an escaped inmate (PowerDMS 2020), and clear calls without patrol officers having to respond (Flemming 2019), respectively. However, there are no systematic, independently validated evaluations on the efficacy of their use.

### Video Surveillance

Law enforcement uses CCTV, also known as video surveillance, primarily to deter crime and record footage for potential future investigations. Today, most cameras are digital, with long-term storage capacity. Cameras can also come with zoom, night-vision, audio, and FRT. Law enforcement agencies have their own CCTV cameras in public places but also may gain access to private cameras through public–private surveillance programs. Oliver & Kugler’s (2021) national survey suggests that just under half of all local police departments and just under 90% of large departments (jurisdictions with 250,000 or more residents) in the United States use video surveillance.

There have been more systematic studies of CCTV than other surveillance technologies covered in this review. Early studies produced mixed results, but a few studies in the United Kingdom attributed significant drops in particular crimes of between 25% and 56%, although displacement effects were not always taken into account (see summary in Slobogin 2007, pp. 84–88). More recent studies are similar in outcome. For instance, one study found that video surveillance alone was effective in reducing vehicle crimes but has not been shown to be associated with a reduction in violent crime (MTAS Res.Inf.Cent.2016).A second study found that cameras in Chicago produced significant crime reductions in high-crime areas but little effect in other areas (Shah & Braithwaite 2013).Another recent summary of research found that video surveillance technology “may help to solve burglaries (Coupe & Kaur 2005) and other crimes (see Ashby 2017), although investigative benefits may be limited by the viewshed of the cameras (see Robin et al. 2020)” (Prince et al. 2021, p. 695). Existing research also suggests CCTV is more effective when combined with improved lighting, security guards, and defensible space (Biale 2008, Cameron et al. 2008, Gerell 2016, Gill et al. 2015, King et al. 2008, Welsh et al. 2015, Welsh & Farrington 2003).

### Facial Recognition

FRT is a form of biometrics that involves matching a human face from a digital image of a video frame against a database of faces. Recent high-profile examples of its use include identifying people during the protests following the death of George Floyd in May 2020 and people involved in the US Capitol attack in January 2021. In developing image databases, police have historically relied on government-provided images, such as mugshots, driver’s licenses, and passport photos (Hill 2021), but increasingly they are resorting to other databases of human face images that come from the public domain or are purchased from private companies (GAO 2021). For example, the Clearview AI database includes more than ten billion images (Fasman 2021,Knight 2021) scraped from the public domain, including Facebook, YouTube, and Venmo. For an American, there is a one in two chance of inclusion in an FBI-accessible database and an even greater likelihood of being in Clearview’s.

Overall, there is little transparency regarding law enforcement’s use of FRT technology. Even some agencies themselves, including those in the federal government, are not certain about whether, or in what ways, FRT is employed (GAO 2021). However, reports indicate Clearview users include approximately 600 law enforcement agencies (Hill 2020, Mac et al. 2020). Furthermore, one source reports that as of September 2021, at least 292 local police departments and 25 state police departments use FRT in some fashion (Fight Future 2021), and Oliver & Kugler’s (2021) nationally representative survey suggests approximately 10% of police departments do so. Although outright moratoria on FRT are relatively rare and can often be evaded through partnerships with neighboring agencies, 19 jurisdictions, including cities such as San Francisco and Boston, and states such as Vermont and Virginia have officially banned police use of FRT (Fight Future 2021) and the European Parliament has called for a ban as well.

Most information regarding the efficacy of facial recognition systems is anecdotal. According to one source, Florida officials recently reported that “they query the system 4,600 times a month” but “only a small percentage of the queries break open investigations of unknown suspects, ”and in California FRT apparently led to one arrest in four years (Valentino-DeVries 2020). Effectiveness is also difficult to measure because “not all successful identifications are logged, and questionable or negative results are not recorded” (Valentino-DeVries 2020). Additionally, even with clear images, the accuracy of FRT across demographic groups is suspect. The most rigorous evaluation to date comes from the National Institute of Standards and Technology (NIST 2019), which found that facial matching algorithms perform less well on very young and very old people, women, and people with darker skin. More specifically, NIST found that false-positive patterns varied across algorithms, but that overall there were higher rates of false positives for one-to-one matching of Asian and African American faces relative to Whites (particularly for African American women) (see also Garvie 2019, Garvie et al. 2016, Haskins et al. 2020, Simonite 2018, Valentino-DeVries 2020). All steps of FRT—including image selection, photo preprocessing, algorithmic search, human analysis of the results of the search, and the investigative follow-up—involve subjectivity.

### Mobile Phone Tracking

According to a recent report by the *New York Times*, “Every minute of every day, everywhere on the planet, dozens of companies, largely unregulated, little scrutinized, are logging the movements of tens of millions of people with mobile phones and storing the information in gigantic data files” (Thompson & Warzel 2019). The location information that cell phones constantly ping is known as cell site location information (CSLI) and GPS data. Depending on the number of apps installed, a cell phone may send data regarding its location thousands of times per day (Dixon 2020).

Law enforcement makes tens of thousands of requests every year to obtain data from cellphone providers, sometimes to obtain real-time location information (the primary focus of this review) but more often to obtain data about a person’s past locations in an effort to determine whether they were near the scene of a crime (a practice sometimes known as geofencing).In fact, “Verizon alone received over 13,000 warrants for cellphone location data in the second half of 2020” (Oliver & Kugler 2021). According to Oliver & Kugler (2021), 75.3% of the departments they surveyed reported using cell phone tracking technology. Law enforcement also purchases data from location companies (Thompson & Warzel 2019).Police departments in larger jurisdictions are more likely to use CSLI to determine locations of people than those in smaller jurisdictions (Marshall et al. 2019). A recent report by Upturn documents the widespread adoption of mobile digital forensics technologies, i.e., cell phone extraction devices to infer location data (in addition to a wide range of other information). The report’s authors filed 110 public record requests to state and local law enforcement agencies and found that more than 2,000 agencies in all 50 states and the District of Columbia have purchased cell phone extraction tools and have performed thousands of extractions, often without a warrant (Koepke et al. 2020).

Given the ubiquity of tracking requests, police departments clearly think tracking is a useful law enforcement technique. There are numerous anecdotal reports that it has aided police in capturing the perpetrators of serious crimes (Foltz v. Comm. 2010, Hubbard 2008, Isikoff 2010, Ohlheiser 2014, United States v. Davis 2014, United States v. Marquez 2010). As with other modalities, however, it is difficult to study the counterfactual of whether an arrest would have been made, and as efficiently, had mobile phone tracking not been available.

### ShotSpotter

ShotSpotter is an acoustic gunshot detection technology. The locators are typically installed at a rate of 20–25 sensors per square mile and primarily connected via 3G and 4G networks (mostly AT&T and Verizon) (SEC 2020).

In 2020, acoustic locator technology was installed in 110 cities and 12 campuses, covering 779 square miles. As of 2021, ShotSpotter evidence had been used in 190 court cases (Burke et al. 2021). The sensors are disproportionately placed in minority communities, which may lead to increased interactions between police and Black people, some triggered by false alerts (Feathers 2021, Illinois v. Williams 2021, Wasney 2017). A recent Associated Press report states ShotSpotter is “usually placed at the request of local officials in neighborhoods deemed to be the highest risk for gun violence, which are often disproportionately Black and Latino communities” (Burke et al. 2021).

The NYU School of Law Policing Project (which receives funding from ShotSpotter) published an evaluation in 2021 that found a significant drop in gun violence in St. Louis, Missouri, after installation of ShotSpotter (Polic. Proj. 2021). However, most studies have found the contrary. One recent study stated that the system “may be of little benefit to police agencies with a pre-existing high call volume,” and found “no reductions in serious violent crimes [while increasing] demands on police resources” (Mares & Blackburn 2020). A second study found that ShotSpotter did not reduce gun violence nor increase community safety (Doucette et al. 2021). Police in Fall River, Massachusetts, have stated that “ShotSpotter worked less than 50% of the time and missed all seven shots in a downtown murder in 2018.” Similarly, the MacArthur Justice Center (2021) studied more than 40,000 dispatches in Chicago and found that 89% of dispatches discovered no gun-related crime and 86% discovered no crime at all; it also reported some evidence that recordings are modified and recoded at the request of police departments (Burke et al. 2021, Drange 2016, Feathers 2021; see also Illinois v. Williams 2021). Charlotte, NC, and San Antonio, TX, have ended their contracts with ShotSpotter because of reliability concerns. Although the company reports a 97% accuracy rate, it has also admitted that “dumpsters, trucks, motorcycles, helicopters, fireworks, construction, trash pickup and church bells have all triggered false-positive alerts, mistaking these sounds for gunshots” (Burke et al. 2021).

### Smart Home Sensors

Police have long relied on ordinary civilians to aid in their surveillance efforts. However, more recently networked technologies and “smart” security devices have intensified this practice of “surveillance deputization” (S. Brayne, S. Lageson, K Levy, L Kilgore, unpublished results). One such example is Amazon Ring. Ring involves a video doorbell that links with other Wi-Fi-enabled products and integrates with its social media app, called Neighbors.

Although precise numbers are unclear, recent reports suggest approximately 2,000 departments are partnered with Ring (Lyons 2021, Lee 2021, Oliver & Kugler 2021). All but two states (Montana and Wyoming) have police and fire departments that partner with Amazon’s Ring system.

An NBC news investigation of 40 law enforcement agencies suggests there is little evidence to support Amazon’s claim that Ring is an effective investigatory tool (Farivar 2020). Thirteen jurisdictions reported that no arrests resulted from reviewing Ring footage, 13 confirmed arrests after reviewing the available data, and 12 jurisdictions “said that they don’t know how many arrests had been made as a result of their relationship with Ring – and therefore could not evaluate its effectiveness” (Farivar 2020).

### See-Through Technology

Several new technologies allow law enforcement to “see” through opaque surfaces. For instance, thermal imagers allow police to detect heat signals through walls, trees, or smoke in real-time and can also enhance the night-vision capabilities of the police (DJI 2018). Imagers can also be used in conjunction with UAVs and CCTV. Other see-through devices rely on electromagnetic signatures, passive millimeter wave technology, or high-frequency radar to detect weapons underneath clothing (Riley 1997, pp. 289–91). See-through technologies are widely used by police departments, and gun detectors are found at every airport and many transportation centers.

The key accuracy issue with these devices is determining what a particular image means. A thermal imager’s identification of a significant heat source inside a building could be evidence of a marijuana farm or evidence of a movie studio. Similarly, even if a gun detector is fairly accurate at detecting a gun, all 50 states and the District of Columbia permit citizens to carry concealed weapons under various circumstances. Thus, although such information may improve police situational awareness when they already suspect an individual of criminal activity, there is no evidence of their utility in solving crimes.

### Communications Surveillance

Classic electronic surveillance consisted of “bugging” a suspect’s phone. Today, wiretapping of phone conversations is supplemented by interception of emails and “envelope” information identifying the sender and receiver of electronic communications (using pen registers and trap and trace devices), and by examination of the content of stored messages and associated subscriber information. Under federal law, the first type of surveillance requires a warrant (which can only be issued if the government shows that other means of obtaining criminal evidence have been tried and failed or are likely to fail), and the second and third types of surveillance also require a court order under most circumstances, although not a warrant [18 U.S.C. § 2510 et seq. (1968)].

The federal government compiles annual statistics about both the frequency and results of electronic surveillance. In 2019–2020, 2,377 wiretaps were authorized, with 1,297 authorized by federal judges and 1,080 authorized by state judges. In 1,503 of these cases, an extension beyond the 30 days of the original order was requested, and all such requests were granted. Most interception orders were focused on mobile phones. As a result of these wiretaps, by December 2020, 6,574 persons had been arrested and 311 persons had been convicted (US Courts 2020). All these numbers were down appreciably from previous years. It has also been reported that, on average, each wiretap intercepts the conversations of more than 100 people, most of them not the target of an investigation or listed on the warrant (see, e.g., US Courts 2011).

As with the other technologies discussed here, the effectiveness of electronic communications technology is hard to gauge. One survey of the available anecdotal evidence suggests that “wiretapping is particularly useful in conspiracies such as organized crime and drug cases” and “foreign-intelligence and terrorism cases” (Landau 2011, p. 98) but also notes that there is a “lack of hard evidence” on its efficacy (Landau 2011, p. 105). Access to telephone records, which has increased exponentially in the past three decades, appears to cut investigation time down substantially. But lack of transparency “leaves no way to determine the effectiveness of the pen registers and trap and traces that the DOJ has been increasingly performing” (Landau 2011, p. 102).

### Stingray

A subset of communications surveillance involves Stingrays, or international mobile subscriber identity (IMSI) catchers. Stingrays essentially operate as fake mobile towers that mimic a wireless carrier cell tower using a stronger signal, which overrides the carrier towers and causes nearby cell phones to connect to them instead. Stingrays can be used to identify and track phones even when they are not making calls or accessing data services. The devices can be mounted in vehicles, airplanes, helicopters, or drones or can be handheld. Typically, law enforcement puts them in cars with compatible computer software (Benway 2018). Documents obtained by the American Civil Liberties Union (ACLU) in 2015 indicate that Stingrays also “have the ability to record the numbers of incoming and outgoing calls and the date, time, and duration of the calls, as well as to intercept the content of voice and text communications” (as cited in Zetter 2020, p. 23). However, the Justice Department asserts that the Stingrays it uses domestically do not intercept the content of communications (Zetter 2020, p. 23).

A 2018 ACLU report shows that at least 26 states are known to have or use Stingrays (Zetter 2020, p. 8). However, the absolute number of police departments with Stingray technology is currently relatively small. Although they had difficulty assessing the prevalence of Stingray use given nondisclosure agreements, Oliver & Kugler (2021) suggest that under six percent of police departments use Stingrays, in part because of their expense and their impact on privacy. The lack of transparency also means there is no systematic data evaluating the efficacy of Stingray technology.

Use of surveillance technologies is increasing among law enforcement agencies. On-the-ground qualitative research involving police, technology companies, and community members is required to understand how surveillance technologies are used and their impact on surveilled community members. Unfortunately, these technologies are often rolled out in secret, without community buy-in, and they are rarely deployed in a systematic fashion. As a result, information about prevalence and effectiveness is limited, as this very brief review illustrates. The National Institute of Justice has recognized that “agencies that plan to deploy and evaluate a new technology would benefit from an end-to-end assessment process that includes collecting comparable baseline data” (NIJ 2017). As many police departments pay for surveillance technology with federal grants, these grants should be contingent upon sharing data, ongoing validation, and independent outcome evaluation.

## SURVEILLANCE LAW

A welter of statutes and regulations at both the federal and state levels governs the use of the surveillance technologies described above. Although some of these laws will be noted below, the primary focus of this analysis will be the Supreme Court’s Fourth Amendment jurisprudence, which sets forth the minimum legal requirements that surveillance by the government must meet. The Fourth Amendment prohibits unreasonable searches and seizures of “persons, houses, papers and effects” and requires that warrants authorizing a search or seizure be based on probable cause and describe with particularity the place to be searched and the person or things to be seized. The Supreme Court has had difficulty in both determining when surveillance is a “search” or “seizure” and deciding whether, if surveillance triggers Fourth Amendment protection, a warrant is required to make it reasonable. As a result, the Fourth Amendment has had little or no impact on most of the surveillance technologies discussed here. Nor in most cases has either Congress or state legislatures seen fit to regulate such surveillance through statutes.

### The Definition of Search

The Fourth Amendment is only implicated by government action that is a search or a seizure. The definition of “search,” the term most relevant to surveillance, has vacillated. The Supreme Court has never defined that word the way a layperson would, as an effort to look at, for, or through something. Rather, in its early cases, the Court determined whether the police action involved physical intrusion into one of the four protected areas listed in the amendment: persons, houses, papers, or effects. Thus, for instance, according to a 1924 Supreme Court decision, police forays onto private property outside the immediate “curtilage” of the home were not searches—even though they involved trespasses—because they did not target the “house” (Hester v. United States, 1924). Nor, the Court held in Olmstead v. United States (1928), did a search occur when police bugged the phone wires outside of the house or when they placed a “detectaphone” against a wall (Goldman v. United States 1942) [although in a later case the Court held that using a “spike mike” inserted beneath the floorboards of a house wall was a search (Silverman v. United States 1961)].

Looking inside a home did not implicate the Constitution either, so long as the observation was from a “lawful vantage point” such as a public sidewalk or an apartment hallway; according to the Court “the eye cannot commit the trespass condemned by the Fourth Amendment” (McDonald v. United States 1948, p. 454). For the same reason, observation of activities that take place entirely in public was not a search. In short, since, by definition, surveillance does not involve physical intrusion, this intrusion-based characterization of Fourth Amendment protection left these types of police actions unregulated by the Constitution.

Then, in 1967, the Court decided Katz v. United States (1967). There, the police bugged a phone booth used by Katz to place bets. Under existing precedent, the government had a seemingly invincible rebuttal to Katz’s argument that the bugging was a search. First, a phone booth is not a person, house, paper, or effect (with the latter term traditionally confined to items of personal property). Second, even if a phone booth were considered a “constitutionally protected area,” the police did not “commit the trespass condemned by the Fourth Amendment,” because the tape recorder did not intrude into the booth or the wires that carried Katz’s conversations. Third, obtaining a recording of those conversations could not trigger Fourth Amendment protection because intangible things like conversations are not only not one of the four protected categories listed in the Fourth Amendment but are also impossible to “seize.”

Yet none of these arguments persuaded a majority of the Court. In holding that the police should have obtained a warrant authorizing the recording, the Court shunted aside the debate over whether a phone booth is a constitutionally protected area. Rather, Justice Stewart’s opinion stated that “the Fourth Amendment protects people, not places” (Katz v. United States 1967, p. 351). Justice Harlan, in his concurring opinion, added that, in defining “what protection [the Amendment] affords to those people. . .the rule that has emerged from prior decisions is a twofold requirement, first that a person have exhibited an actual (subjective) expectation of privacy and, second, that the expectation be one that society is prepared to recognize as ‘reasonable’” (Katz v. United States 1967, p. 361).

The reasonable expectation of privacy language in Justice Harlan’s opinion has since become the primary means of defining when law enforcement engages in a Fourth Amendment “search.” Like the pre-*Katz* definition of search, this new formulation is narrower than the lay definition of that word. But, given the resolution of the *Katz* case, the expectations-of-privacy language seemed to contemplate a much broader scope of Fourth Amendment protection than the trespass-oriented language found in earlier Court opinions.

Congress certainly read *Katz* that way. In 1968—one year after *Katz* and a second Supreme Court decision, Berger v. New York (1967), made clear that wiretapping is a Fourth Amendment search that requires a warrant—Congress enacted Title III of the Omnibus Crime Control and Safe Streets Act [18 U.S.C. § 2510 et seq (1968)]. As noted above, Title III, as it has come to be called, imposed significant restrictions on electronic eavesdropping that went beyond those applied to a traditional warrant. Whereas a traditional warrant can be issued by a justice of the peace, Title III requires that electronic surveillance warrants be issued by a district court judge. Furthermore, such a warrant may be issued only if the judge finds that “normal investigative procedures have been tried and failed or reasonably appear to be unlikely to succeed if tried or to be too dangerous,” a finding that is not required for a traditional warrant [18 U.S.C. § 2518(3) (1968)]. Also, interceptions of communications are to be “conducted in such a way as to minimize the interception of communications not otherwise subject to interception” [18 U.S.C. § 2518(4) (1968)]; traditional warrants must describe with particularity the thing to be seized but do not include a minimization limitation.

*Katz* and Title III signaled that some types of surveillance would be subject to heightened scrutiny. Despite *Katz*’s relatively capacious language, however, for the next half-century the Supreme Court’s caselaw had the effect of confining that decision’s holding to its facts, with the result that surveillance that did not involve interception of communications was left unregulated by the Constitution. This caselaw developed four doctrines—the knowing exposure doctrine, the general public use doctrine, the evidence-only doctrine, and the assumption of risk doctrine—that had the effect of freezing Fourth Amendment law in its pre-*Katz* state throughout the twentieth century. Only in the past twenty years has the Court, perhaps realizing that its jurisprudence had essentially rendered the Fourth Amendment irrelevant in the digital age, begun to back away from all four doctrines.

#### Knowing exposure.

*Katz* itself said that although conversations over a public phone can be private for Fourth Amendment purposes, “[w]hat a person knowingly exposes to the public, even in his own home or office, is not a subject of Fourth Amendment protection” (Katz v. United States 1967, p. 351). The Supreme Court confirmed this notion in several post-*Katz* cases. In 1983, it held that “[a] person traveling in an automobile on public thoroughfares has no reasonable expectation of privacy in his movements from one place to another” (United States v. Knotts 1983, p. 281). In three subsequent cases, the Court relied on the same notion in holding that police could use a plane to spy on private property without implicating the Fourth Amendment; after all, the Court stated, “any member of the public in navigable airspace” could have seen what the police saw and no physical intrusion onto the property was involved (California v. Ciraolo 1986, p. 213; Dow Chemical v. United States 1986; Florida v. Riley 1989). Under a strict reading of these cases, any surveillance of the public streets, whether by CCTV, ALPRs, Ring, ShotSpotter, satellites, or drones, does not implicate the Fourth Amendment nor does the use of cellphone tracking technology that only picks up public travels. FRT that scans faces of pedestrians would also evade Fourth Amendment strictures.

In 2012, however, the Supreme Court took a cautious step in the other direction. In United States v. Jones (2012), the Court held that planting a GPS device on a car and using it to track Jones’s car was a Fourth Amendment search. That holding suggested that some types of public surveillance are subject to constitutional regulation.

At the same time, the majority in *Jones* was careful to limit its decision to the use of a GPS covertly placed on the car—an action the Court termed a trespass. Harkening back to pre-*Katz* days, this is a very narrow holding, limited to when the tracking device works a physical intrusion on private property. Thus, real-time tracking relying on cellphone signals does not trigger *Jones*, nor does use of a radio frequency identification device that communicates current and past routes to an intelligent transportation system computer (Majoras et al. 2005), because the transponder is placed in the car before the owner buys it.

However, it is also important to note that five justices in *Jones* indicated they would be willing to consider GPS tracking that did not involve a trespass a search, at least if it were “prolonged.” Picking up on this cue, several lower courts have held that this type of tracking is a search (see summary in United States v. Howard 2019). A few lower courts have even held that any type of prolonged technologically enhanced surveillance (e.g., with CCTV cameras) is a search, although to date most courts have held to the contrary (see summary in United States v. Tuggle 2021).

#### General public use.

The second Supreme Court doctrine construing the reach of the Fourth Amendment looks at whether any technology used by the government is “in general public use;” if so, no search occurs, even if the surveillance is of the inside of the home. In 1986, the Supreme Court faced a Fourth Amendment claim brought by Dow Chemical Company, arguing that use of a $22,000 mapmaking camera to spy on its property from the air was a Fourth Amendment search (Dow Chemical v. United States 1986). Recognizing the knowing exposure doctrine might spell doom for such a claim, the company emphasized that the mapmaking camera was a specialized device that the typical individual was unlikely to have. But the Court demurred, declaring that such cameras are “generally available to the public” and thus police needed no justification to use them (Dow Chemical v. United States 1986, p. 238).

Fifteen years later, however, the Court appeared to rethink the general public use doctrine. In Kyllo v. United States (2001) it held, contrary to what one might have expected from *Dow Chemical*, that a thermal imaging device costing only about $10,000 is not in general public use. Therefore, the Court concluded, relying on it to ascertain heat differentials inside a house is a search, at least when such information “would. . .have been unknowable without physical intrusion” (Kyllo v. United States 2001, p. 40).

As with its treatment of the knowing exposure doctrine in *Jones*, however, the Court’s decision in *Kyllo* was cautious. The decision did not reject the general public use doctrine, and in fact in dictum it specifically recognized its continued significance. So presumably police can use any item that is easily purchasable, including binoculars, telescopes, or Startrons (night-vision binoculars that can be purchased for about $200) without triggering the Fourth Amendment. Less clear is whether innovative surveillance technologies such as drones or facial imaging implicate the Fourth Amendment; given the ubiquity of both drones and facial recognition functions (on cellphones), however, the general public use doctrine may exempt from constitutional regulation those surveillance techniques as well.

Note also that, whereas *Dow Chemical* involved surveillance of the outside of business property, *Kyllo* permits surveillance of the home with technology if that technology is available to the general public. Furthermore, *Kyllo* states that even sophisticated technology—devices that are not in general public use—can be used to look inside a home if it only detects what a police officer could see with the naked eye from a traditional “lawful vantage point” (Kyllo v. United States 2001, p. 36). That means that, even if the technology is not in “general public use,” police can, without worrying about the Fourth Amendment, use satellite and drone cameras to look into a picture window, if all they detect is something an officer who happens to be passing by could see from the sidewalk.

#### Evidence only.

Even those parts of the home that are curtained off may not be protected from sophisticated technological surveillance if the technology is evidence-only, meaning that it detects only items that are evidence of criminal activity. This idea—the third Supreme Court doctrine defining reasonable expectations of privacy—was first broached in a case involving a drug-sniffing dog, where the Court concluded that “government conduct that can reveal whether [an item is contraband] and no other arguably ‘private’ fact. . .compromises no legitimate privacy interest” (Illinois v. Caballes 2005, p. 408, relying on United States v. Place 1983). Some scholars have called this a “binary search” because it tells police whether, and only whether, an item of interest is present (see, e.g., Rosenthal 2014).

As noted earlier, scientists have developed “mechanical dogs” that can sniff out weapons or contraband. Most of these instruments, particularly if based on x-ray technology, are not evidence-specific; they expose other items as well. But, as noted above, some companies purport to have designed devices that can detect only weapons, which might fit this definition (Smart Solut.2021). If an evidence-only device were developed, it would allow police to cruise the streets scanning cars, people, and homes for illicit items without infringing on Fourth Amendment interests because, by definition, it only reveals evidence of crime or potential threats.

This doctrine too may be in transition after the Supreme Court’s decision in Florida v. Jardines (2013). There, the Court held that a dog sniff of a home, from the sidewalk leading up to it, is a search. However, as with its treatment of the knowing exposure doctrine in *Jones*, the Court limited this holding to situations in which there is a physical intrusion on private property or a physical detention. Furthermore, if the predicate intrusion is lawful—for instance, a person has been lawfully stopped at a checkpoint or for a traffic violation—that obstacle to an evidence-specific search disappears. Thus, even after *Jardines*, evidence-only searches do not, in and of themselves, implicate the Fourth Amendment.

This conclusion is particularly important because of the huge increase in “alert” technology in the past few years made possible by high-frequency real-time data collection sensors. Whereas query-based systems operate in response to a user query, such as when an officer runs a license plate during a traffic stop, in alert-based systems, users receive real-time notifications when certain variables or configurations of variables become present in the data (Brayne 2018, 2021). The most prominent examples are facial recognition and license plate reader systems that match faces or licenses to most-wanted or “hot” lists. If these systems function as advertised, they might be classified as evidence-only (and thus ungoverned by the Fourth Amendment) if they alert only to persons or cars that have been shown to be associated with crime.

One objection to that analysis is that, as discussion above noted, these technologies are not always accurate and therefore are not truly binary. Yet in Florida v. Harris (2013), the Court discounted a similar argument made against reliance on drug-detection dogs, which also are sometimes mistaken. There, the Court held that “[t]he question—similar to every inquiry into probable cause—is whether all the facts surrounding a dog’s alert, viewed through the lens of common sense, would make a reasonably prudent person think that a search would reveal contraband or evidence of a crime” (Florida v. Harris 2013, p. 248). Strongly hinting that a dog that has successfully completed training can be used to detect drug odors, *Harris* made clear that the state need not prove that its evidence-only technique is perfect or even close to it, only that it has been certified through testing.

#### Assumption of risk.

Although the first three doctrines exempt a wide swath of surveillance technologies from Fourth Amendment protection, their impact pales in comparison to the impact of the fourth doctrine defining when expectations of privacy are reasonable. In a series of decisions beginning in the 1960s, the Supreme Court has held that people assume the risk that information disclosed to others will be handed over to the government and thus cannot reasonably expect it to be private; to put this in legalese, individuals (the first party) cannot prevent the government (the second party) from obtaining personal information that is voluntarily surrendered to another person or entity (the third party), which explains why this rule is also often called the “third-party doctrine.”

The most important decision in this regard is Miller v. United States (1976). In Miller (1976, p. 443), the Court held that an individual “takes the risk, in revealing his affairs to another, that the information will be conveyed by that person to the government. . .even if the information is revealed on the assumption that it will be used only for a limited purpose and the confidence placed in the third party will not be betrayed.” In reaching this conclusion, the Court relied on earlier cases in which the third-party confidant was a person (a friend or acquaintance) (see, e.g., Hoffa v. United States 1966). In *Miller*, in contrast, the third party was a bank. The Court held that even here one assumes the risk of a breach of confidence and, therefore, that depositors cannot reasonably expect that the information they convey to their banks will be protected by the Fourth Amendment. In Smith v. Maryland (1979, p. 743), the Court held the same thing with respect to phone numbers maintained by a phone company for billing purposes, stating that because telephone users “know that they must convey numerical information of the phone company; that the phone company has facilities for recording this information; and that the phone company does in fact record this information for a variety of legitimate business purposes. . .it is too much to believe [that users] harbor any general expectation that the numbers they dial will remain secret.”

The decisions in *Miller* and *Smith*, which came at the dawn of the Information Age, have enormous implications for law enforcement investigation today. The quantity of the world’s recorded data has doubled every year since the mid-1990s (Holst 2021). The computing power necessary to store, access, and analyze data has also increased exponentially over the past decades and at increasingly cheaper costs (Seifert 2007). Virtually every aspect of our lives sits on a third-party computer somewhere. In the old days, accumulating data from disparate sources involved considerable work and several days, if it was possible at all; today, it can often occur at the touch of a button, with the result that private companies as well as governments excel at creating what Daniel Solove has called “digital dossiers” (Solove 2003).

Once again, however, there are signs that the Supreme Court is starting to realize what it has done. In 2018, the Court decided Carpenter v. United States (2018). Carpenter was suspected of involvement in a series of store robberies. Police accessed over 130 days of CSLI from Carpenter’s two wireless carriers to see if Carpenter was near the stores when they were robbed and discovered that Carpenter (or at least his phone) was in proximity to at least four of the stores on the relevant days. Although the police had a court order to access the CSLI data, that order was not a full-blown warrant based on probable cause. So Carpenter argued that accessing his phone records was an illegal search.

In response, the government relied on *Miller* and *Smith*. Carpenter, the government argued, knew or should have known that his location information was maintained by his common carrier; therefore, Carpenter assumed the risk that the carrier would turn that information over to law enforcement. That type of argument would have won if the Court had adhered to the holdings in *Miller* and *Smith*. But the Court instead held that the police should have obtained a warrant. Chief Justice Roberts, who wrote the majority opinion, gave several reasons for the holding but two in particular stand out: (*a*) “[t]here is a world of difference between the limited types of personal information addressed in *Smith* and *Miller* and the exhaustive chronicle of location information casually collected by wireless carriers today”(Carpenter v. United States 2018,p.2219) and (*b*) “[c]ell phone location information is not truly ‘shared’ as one normally understands the term [because] cell phones and the services they provide are. . .indispensable to participation in modern society” (Carpenter v. United States 2018, p. 2220).

Although *Carpenter* cut against precedent, neither rationale was a complete surprise. The first rationale resonated with the opinions of five justices in *Jones*. Recall that the majority opinion in that case focused on the trespass issue. But in his concurring opinion, Justice Alito, joined by three others, argued that Jones should have won even had there been no trespass, given the prolonged nature of the tracking that occurred (28 days). In a separate concurring opinion, Justice Sotomayor, while joining the majority’s trespass reasoning, likewise expressed concern about the “aggregated” data that tracking devices allow (United States v. Jones 2012, p. 416). So five justices fastened on the government’s ability to amass significant quantities of information as a reason for triggering the Fourth Amendment. Four years before *Carpenter*, another Supreme Court case, Riley v. California (2014) overturned centuries of precedent permitting warrantless searches of personal effects found on an arrested person, when it held that search of an arrestee’s cell phone requires a warrant; resonating with *Jones* and presaging *Carpenter*, the Court stated that comparing a search of an arrestee’s wallet or purse to search of a phone “is like saying a ride on horseback is materially indistinguishable from a flight to the moon” (Riley v. California 2014, p. 393).

The second rationale proffered by Chief Justice Roberts for the result in *Carpenter* rightly called out as fiction the notion that we “voluntarily” transfer information to our phone companies. This was a recognition by the Court that people have to provide information to third-party entities—ranging from phone companies to banks to Internet browsers—to function in modern society. The possible implication of this reasoning is that the third-party doctrine is on its last legs.

Again, however, as it has done with respect to the other doctrines defining “search,” the majority in *Carpenter* emphasized that it was limiting its decision to the facts of *Carpenter*. Thus, although a warrant is required to obtain a large amount of CSLI data, as a constitutional matter a subpoena or even a mere request from the police might still be all that is needed to obtain other types of personal information collected and maintained by third parties. The same question arises in connection with geofencing, in which the government requisitions from Google and other providers information about phone numbers near the scene of a crime with the aim of identifying perpetrators or eyewitnesses, a practice that is becoming increasingly popular (Whittaker 2021). Some courts have required a warrant before police access such information (which effectively precludes geofencing unless the police already have a good idea of who the perpetrator is), whereas others, pointing to the fact that only a small amount of information about a single individual is revealed (e.g., their location over a short period of time), require little or no justification (see Slobogin 2022).

*Carpenter*, and the assumption of risk doctrine more generally, involve government access to records of past activity, and thus neither is directly relevant to the law of surveillance, which this review is defining as investigations in real-time. Nonetheless, the decision is likely to have a significant impact on judicial analysis of surveillance. For instance, relying on *Carpenter*, the Fourth Circuit Court of Appeals held, in Leaders of a Beautiful Struggle v. Baltimore (2021), that an aerial surveillance program run by the city was unconstitutional. Under the program, the aerial cameras only captured people as blurry dots and the recordings were accessed only when police wanted to determine where individuals who were near the crime scene at the time it occurred came from or subsequently went. Nonetheless, emphasizing the fact that the camera recordings were retained for 45 days and could conceivably be used to trace a person’s daytime travels during that period, the Fourth Circuit held that the program violated *Carpenter*, which it concluded stood for the proposition that “prolonged tracking. . .invades the reasonable expectation of privacy that individuals have in the whole of their movements”(Leaders of a Beautiful Struggle v. Baltimore 2021, p. 341).

As the dissent in Leaders of a Beautiful Struggle (2021, p. 353) points out, this line of reasoning calls into question all surveillance systems, including CCTV, ALPRs, and GPS. The Fourth Circuit appeared to hold that any system that has the capability of permitting long-term tracking implicates the Fourth Amendment, even if that capability is only used under limited circumstances. If that is to be how *Katz* and its progeny are applied to surveillance technologies, then not only the assumption of risk doctrine but also the knowing exposure and general public use doctrines are clearly on their way out (and the evidence-only doctrine may be rendered moot to the extent governments are prohibited or deterred from establishing CCTV and other systems on which some binary surveillance technologies rely).

It is also important to recognize, however, that a conclusion that use of a surveillance technology is a search does not end the analysis. “Reasonable” searches are still permissible. That is the second question the Fourth Amendment poses.

### The Definition of Reasonableness

Typically, a search is reasonable if it is authorized by a warrant based on probable cause. In *Jones*, *Kyllo*, *Jardines*, and *Carpenter*, the Supreme Court indicated that a warrant was required to authorize the police action (although in *Jones* the majority never came right out and said so). Likewise, in *Leaders of a Beautiful Struggle*, the Fourth Circuit held that a warrant was required for aerial surveillance. It is important to recognize, however, that there is a difference between the first four cases and the last one. In each of the Supreme Court’s cases, the police could have obtained a warrant, at least in theory, because police had an identified suspect. In *Leaders*, in contrast, a warrant would have been impossible to obtain—probable cause obviously could not be demonstrated for every person subject to surveillance, and even after a crime occurred, probable cause to track a particular “dot” could not be made out unless, perhaps, there was only one dot at the crime scene.

The Supreme Court’s cases suggest that when, as in *Leaders*, the reasonableness of a program (as opposed to a search of a particular individual) is at issue, “an adequate substitute for a warrant” may suffice. This language comes from the Court’s decision in Donovan v. Dewey (1981, p. 603), which dealt with the constitutionality of a statute authorizing inspections of coal mines for dangerous conditions. Despite the fact that the government could not demonstrate suspicion with respect to any particular coal mine before an inspection, the Court upheld the searches authorized under the statute, because it

Requires inspection of all mines and specifically defines the frequency of inspection. . . .[T]he standards with which a mine operator is required to comply are all specifically set forth in the [Mine Safety] Act or in. . .the Code of Federal Regulations. . . .[R]ather than leaving the frequency and purpose of inspections to the unchecked discretion of Government officers, the [program] establishes a predictable and guided federal regulatory presence (Donovan v. Dewey 1981, pp. 603–4).

In other words, because the program was legislatively authorized, established clear criteria for when inspections were permitted, and required that all mines be inspected—thus minimizing the potential for arbitrary enforcement—it did not violate the Fourth Amendment.

The Court made the same type of point in upholding a checkpoint for illegal immigrants near the border with Mexico in United States v. Martinez-Fuerte (1976).The checkpoint in that case was set up by higher-level authorities, and everyone who came to the checkpoint was subject to initial seizure. Similarly, in a case holding that random car license checks made at the behest of individual officers are unconstitutional, the Court noted in dictum that a license checkpoint that stopped everyone or every third or fifth person would pass constitutional muster (Delaware v. Prouse 1979). Applied to the aerial surveillance system in *Leaders*, these cases would not require a warrant to set up the drone surveillance system but would mandate that the system be legislatively authorized and directed at the entire population or implemented in some other neutral, even-handed fashion.

The Court has also sent signals about government’s obligations governing both the accuracy and the retention of information collected through a surveillance program. With respect to accuracy obligations, in Herring v. United States (2009, pp. 146–47), the Court suggested, without holding, that evidence found during an arrest based on an expired warrant should be excluded if the defendant can demonstrate the presence of “routine” and “widespread” errors in the arrest database; thus, a demonstration of systemic database errors might implicate the Constitution. On a subconstitutional level, the Court’s decision in Daubert v. Merrell Dow Pharmaceuticals (1993), which has been followed in more than half the states, requires that under the rules of evidence the accuracy of technology used in the adjudication process be subject to a testing and verification process. If applied to many surveillance techniques (see, e.g., the earlier discussion of ShotSpotter), *Daubert* might pose a barrier to their use (see Hu 2015).

With respect to the retention issue, in Whalen v. Roe (1977, p. 605), the Supreme Court noted with favor the fact that a state statute governing collection of prescription drug information prohibited unwarranted disclosures to the public, a protection it said “arguably has its roots in the Constitution” in some circumstances; in later cases, the Court “assumed, without deciding, that the Constitution protects a privacy right of the sort mentioned in *Whalen*. . .” (NASA v. Nelson 2011, p. 138). Similarly, in Maryland v. King (2013), in upholding compelled DNA sampling of arrestees, the Court emphasized that the state was prohibited from using the samples for any purpose other than identification. Applied to surveillance systems, these cases require that authorizing legislation or regulations establish standards governing security and retention of the data. For instance, a statute authorizing a CCTV system might stipulate that the recordings may only be used for the purpose of investigating serious crimes and must be destroyed within three days unless they are needed for such an investigation (see US Dep.Homel.Secur.2007). Law enforcement surveillance efforts might also be affected by laws such as California’s Consumer Privacy Act [Cal. Civil Code, § 1798.100 et seq. (2020)] and Illinois’s Biometric Information Privacy Act [Ill. Stat. 740 § 14/5 et seq. (2008)], which give individuals the right to have their personal data—including images collected for facial recognition purposes—deleted from private company data systems (although there are numerous exceptions to the general rule).

Assuming a surveillance system is permissible under these types of rules, using it to target a particular individual would be governed by cases like *Jones*, *Kyllo*, and *Carpenter*. What remains unclear is whether the Court will maintain its distinction between prolonged and short-term surveillance and, if so, whether short-term surveillance will be entirely exempt from Fourth Amendment protection or instead be subject to some type of constitutional regulation. For instance, the Court has been willing to recognize that some searches and seizures that it considers to be relatively un-intrusive can be justified on a lesser showing, which it calls “reasonable suspicion” (New Jersey v. T.L.O. 1985, Terry v. Ohio 1968). Perhaps that caselaw will eventually be applied to cases involving short-term surveillance of suspects.

### The State Action Requirement

For an intrusion to trigger Fourth Amendment protection it must not only be a search or a seizure, it must also involve “state action,” because the Fourth Amendment (and the other rights in the Bill of Rights) only regulate actions of the government, not of private individuals. In upcoming years, the scope of the state action requirement may be the most important legal issue with respect to surveillance. Today, private companies, many of them data brokers, have acquired mountains of information about each of us and are willing to monetize it—indeed, they may be set up with that goal in mind (Ohm 2012, Ferguson 2017), with some relying on metered pricing models that give law enforcement agencies logged access for individual queries (Brayne 2021). If the government can obtain personal information by announcing it is willing to pay for any data or surveillance images showing wrongdoing that nongovernmental actors can access without implicating the state action requirement, it could well do an end run around all the Fourth Amendment restrictions described previously.

The Supreme Court has found state action not only when government directs a private party to conduct a search but also when there are “clear indices of the Government’s encouragement, endorsement and participation” (Skinner v. Railway Association 1989,pp.615–16).Such indices might be present when surveillance data are sought from companies that collect them for profit, even in the absence of an explicit government direction that the company obtain it. As Kiel Brennan-Marquez (2018) has noted, many of these companies are repeat players, their existence depends in large part on government largesse, and their technological capabilities enable them to access the information easily. Given the monetary incentives, they may even be tempted to engage in intrusions that a government agent would not be authorized to undertake.

If, in light of these realities, it were concluded that routine government use of a given company’s surveillance information constitutes state action, the implications could be significant. It would require not only that, before acquiring information from such companies, the government have the requisite justification for accessing the type of information it wants but also that the data collection or surveillance by the company meets the same legislative authorization and data security and retention requirements that Fourth Amendment doctrine imposes on government-run programs.

## CONCLUSION

Despite their ubiquity, the empirical evidence base for surveillance technologies is weak. For most technologies, even basic information about how many departments use them or how they are deployed is lacking. Even less accessible is good information about surveillance technology’s financial and privacy costs, its impact on police–community relations, and its value in detecting and deterring crime.

Although there is relative consensus regarding the lack of empirical data on many surveillance technologies, there is a distinct lack of consensus about what to do about it. Whereas reformist approaches tend to focus on using existing constitutional, statutory, and evidentiary frameworks to regulate the police, abolitionists argue that even well-intentioned reform efforts have the ultimate effect of legitimizing and entrenching the carceral system and creating new pathways for introducing surveillance technologies in other jurisdictions and contexts. Scholars, activists, policymakers, and lawyers have variously argued that given some combination of the lack of evidence about the effectiveness of these technologies, their intrusiveness, and the possibility that they will have an unfairly disparate impact on minority communities, they should be banned altogether or at least significantly circumscribed, through either executive action, legislative direction, or constitutional litigation.

To date, the courts and most legislatures have avoided this debate. At the same time, the courts have begun to recognize the extent to which technology has enhanced police ability to conduct surveillance. Increasingly, they appear willing to interpret Fourth Amendment search doctrine to encompass new pervasive surveillance techniques and perhaps even to regulate those techniques that are primarily instigated by private parties. The Fourth Amendment is most likely to require a warrant when surveillance technologies are used to observe activities inside the home, access the contents of communications and cellphones, and track public activities over a long period of time. If instead government uses technology to collect information for later access, courts are more likely to defer to statutory and administrative regulation of the data collection and its retention (Slobogin 2022). As technology continues to lure police agencies, legislatures must step in to fill gaps in the constitutional regime by ensuring a systematic, cost-effective, transparent, and accountable approach, both through funding and reviewing research and through leveraging the power of the purse.
